# Performance of adiposity indicators in predicting metabolic syndrome in older adults

**DOI:** 10.20945/2359-3997000000372

**Published:** 2021-04-27

**Authors:** Luana Ferreira Alves, Jeofran Oliveira Cruz, Adriana Lucia da Costa Souza, Carolina Cunha de Oliveira

**Affiliations:** 1 Universidade Federal de Sergipe Lagarto SE Brasil Graduação em Nutrição, Universidade Federal de Sergipe, Lagarto, SE, Brasil; 2 Universidade Federal de Sergipe Departamento de Nutrição Lagarto SE Brasil Departamento de Nutrição, Universidade Federal de Sergipe, Lagarto, SE, Brasil

**Keywords:** Metabolic syndrome, body composition, anthropometry, adiposity, aging

## Abstract

**Objective:**

To evaluate the association between some indicators of adiposity and markers of metabolic disorder, evaluate their performance in predicting metabolic syndrome (MetS), and identify their cutoff values among older adults, both in the overall sample and according to sex.

**Subjects and methods:**

Cross-sectional study in 159 older men and women. MetS was defined according to the harmonized criteria. The assessments included waist circumference (WC), waist-to-height ratio (WHtR), conicity index (C index), lipid accumulation product (LAP), visceral adiposity index (VAI), body mass index (BMI), A body shape index (ABSI), area under the receiver operating characteristic curve (AUC), sensitivity, and specificity.

**Results:**

LAP and WHtR resulted in the largest AUC values (>0.80). In both sexes, the best indicators were LAP, WC, and WHtR. Both LAP and WHtR presented the highest Youden's index values in the overall sample, with cutoff values of approximately 46.9 (sensitivity 75.0%, specificity 76.7%) and 0.56 (sensitivity 79.3%, specificity 69.8%), respectively. When analyzed by sex, BMI, WC, WHtR, and LAP yielded the highest Youden's index values for the prediction of MetS in older women.

**Conclusion:**

The indicators LAP, WC, and WHtR performed well in identifying the presence of MetS in older women and could be used to individually or collectively assess and monitor MetS.

## INTRODUCTION

Metabolic syndrome (MetS) can be characterized as the occurrence of abdominal obesity, dyslipidemia, hyperglycemia, and borderline blood pressure (BP) levels. It is considered one of the greatest public health challenges worldwide since it is directly associated with an increase in risk factors for cardiovascular disease and type 2 diabetes mellitus (1).

In Brazil, as in other countries, representative data on the prevalence of MetS is scarce, ranging from 10.7% to 40.5% in different populations (2), with this prevalence tending to increase with advancing age. For early identification of metabolic disorders, studies suggest the use of body measurements to assess abdominal adiposity since the increase in these parameters is associated with metabolic and cardiovascular abnormalities (3–5).

In addition to assessing classic adiposity indicators, such as body mass index (BMI) and waist circumference (WC), studies have also assessed other indicators to predict MetS in older adults, including the lipid accumulation product (LAP), visceral adiposity index (VAI), waist-to-height ratio (WHtR), a body shape index (ABSI), and conicity index (C index) (3–7). However, these indicators are rarely discussed in the literature, especially in older populations.

With the objective of increased efficiency and due to their simplicity, speed, and functionality, the use of indicators to predict MetS can facilitate the identification of this syndrome in clinical practice. There is currently a lack of consensus on the diagnostic criteria for MetS and a single definition for all populations; this occurs due to functional characteristics and different cutoff values for diagnostic criteria, many of which are specific for adolescents and young adults (1,6,8,9).

Considering that older adults have a higher prevalence of cardiovascular events, parameters ensuring early diagnosis of MetS are essential. Therefore, the objective of the present study was to evaluate the relationship of some indicators of adiposity with markers of metabolic disorder, evaluate the performance of some adiposity indicators in predicting MetS, and identify their cutoff values in older adults analyzed as an entire sample and categorized by sex.

## SUBJECTS AND METHODS

### Study design and sample

This cross-sectional study was carried out in older adults of both sexes, seen at a geriatric outpatient clinic in the city of Lagarto (Sergipe, Brazil). The protocol of the study was approved by the Research Ethics Committee of the Federal University of Sergipe and was registered with CAAE number 25414314.2.0000.5546. The study was conducted in accordance with the Declaration of Helsinki.

Sociodemographic data, including age and sex, were collected. The following inclusion criteria were adopted: age 60 years or older, any social class, ability to walk, and agreement to participate in the study by signing a free and informed consent form. The criteria for non-inclusion were physical and/or postural limitations precluding anthropometric measurements, cognitive limitations, swelling, ascites, and/or visceromegaly.

### Anthropometric evaluation

The parameters evaluated were weight, knee height (KH), and WC. The measurements were obtained following the standard techniques proposed by Lohman and cols. (10). Height was estimated from KH according to the equation proposed by Chumlea and cols. (11). WC was measured at the midpoint between the last rib and the iliac crest during exhalation, according to the World Health Organization (WHO) criteria (12).

### Laboratory evaluation

Results of laboratory tests (lipids and glycemia) obtained up to 1 month from the data collection (or requested by a geriatrician) were retrieved from the participants’ medical records. Normal results were based on the criteria established by the Updated Brazilian Guideline on Dyslipidemias and Atherosclerosis Prevention (13) and the Brazilian Diabetes Society Guidelines (14): total cholesterol (TC) < 190 mg/dL, high-density lipoprotein cholesterol (HDLc) > 40 mg/dL for men and > 50 mg/dL for women, low-density lipoprotein cholesterol (LDLc) < 130 mg/dL, triglycerides < 150 mg/dL, non-HDLc < 160 mg/dL, and glycemia < 90 mg/dL. The TC/HDLc and LDLc/HDLc ratios were calculated (15).

### Adiposity indicators

The adiposity indicators evaluated were BMI, WC, C index, WHtR, ABSI, VAI, and LAP. We chose these adiposity indicators for the present study because they include traditional and clinically relevant markers and have been analyzed in a few studies in older adults, especially in Brazil, thus allowing a comparison.

The C index was obtained using weight, height, and WC measurements and applying the equation proposed by Valdez and cols. (16), *i.e.*, C index = WC in m / 0.109 x √(weight in kg) / height in m. WHtR was calculated by dividing WC by height, both in centimeters (17), and BMI was obtained by the following equation, according to the WHO: weight in kg/height in m² (18). ABSI was calculated with the equation proposed by Krakauer & Krakauer (19) using WC, BMI, and height values, as follows: ABSI = WC in m/(BMI in kg/m²)^2/3^ × height in m^1/2^). VAI was calculated using the formula proposed by Amato and cols. (20), for men VAI = {WC in cm/[39.68 + (1.88 × BMI in kg/m²)]} × (TG in mmol/L/1.03) × (1.31/HDLc in mmol/L) and for women VAI = {WC in cm/[36.58 + (1.89 × BMI in kg/m²)]} × (TG in mmol/L/0.81) × (1.52/HDLc in mmol/L). LAP was calculated according to Kahn's proposal (21), in which the equation for men is LAP = (WC in cm – 65) × TG in mmol/L and for women is LAP = (WC in cm – 58) × TG in mmol/L.

The concentrations of HDLc and TG were converted from milligrams per deciliter (mg/dL) to millimoles per liter (mmol/L) and divided by the constants 0.02586 and 0.01129 mmol/L, respectively, to calculate VAI and LAP.

### Diagnosis of metabolic syndrome

The diagnosis of MetS was based on the harmonized MetS criteria (22). Therefore, an individual should present at least three of the four following components: WC (for South American populations) ≥ 80 cm women and ≥ 90 cm for men; dyslipidemia (HDLc ≤ 40 mg/dL for men and ≤ 50 mg/dL for women or TG ≥ 150 mg/dL, or specific treatment); BP (systolic BP [SBP] ≥ 130 mmHg or diastolic BP [DBP] ≥ 85 mmHg or specific treatment), and glycemia (≥ 100 mg/dL, or specific treatment).

### Statistical analysis

The analysis was performed using the software SPSS, version 20.0 (IBM Corp., Armonk, NY, USA). The variables characterizing the sample were expressed in measurements of central tendency and dispersion, including analyses stratified by sex.

The normality of the distribution of quantitative data was tested using the Kolmogorov-Smirnov test. To compare mean values between variables, Student's *t* test was used for data with normal distribution, and the Mann-Whitney test for data with nonparametric distribution. Mean values were calculated to present data pertaining to markers of metabolic disorder and obesity between sexes, revealing the participants’ profiles. Correlations between variables were performed using Pearson's correlation for variables with normal distribution and Spearman's correlation for those with a non-normal distribution. This correlation analysis was performed to identify relationships between indicators of adiposity and markers of metabolic disorder, as proposed in the study's aims.

Receiver operating characteristic (ROC) curve was used to determine the best cutoff values for adiposity indicators in diagnosing MetS in older adults. Using ROC curves, the area under the curve (AUC) can be used to quantify how well a predictor discriminates individuals with from those without a disease (23), in this case, MetS. The AUC and respective 95% confidence intervals (95% CIs) were then calculated to determine the most sensitive and specific cutoff values for adiposity indicators in the study population. An AUC value equal to 1, or close to this value and greater than 0.80, indicates a perfect prediction, and an AUC of 0.5 indicates the absence of predictive power (24). Sensitivity, specificity, Youden's index, positive predictive value (PPV), negative predictive value (NPV), and their respective cutoff values, with a more appropriate balance between them, were examined. The optimal cutoff values were defined as the points on the ROC curve where the Youden's index (sensitivity + specificity – 1) was highest. A significance level of 5% was adopted for all tests.

## RESULTS

The study included a sample of 159 older adults with a mean age of 70.9 ± 7.4 years and comprising mostly women (50.3%). The prevalence of MetS was 73% (57% among men and 88.8% among women). In terms of markers of metabolic disorder, women had higher mean values of non-HDLc, TC/LDLc, and LDLc/HDLc, while HDLc was higher in men (p < 0.05). Regarding adiposity indicators, mean WC and C index values were higher in men, while mean BMI, WHtR, LAP, and VAI values were higher in women (p < 0.05) (Table 1).

**Table 1:** Descriptive analysis of markers of metabolic disorder and adiposity indicators according to sex in older adults (n = 159) seen at a geriatric outpatient clinic in the city of Lagarto (Sergipe, Brazil)

Variables	Total sample (n = 159)	Men (n = 79)	Women (n = 80)	p
**Markers of metabolic disorder**								
Glucose (mg/dL)	99.00 (91.00-121.00)	99.00 (90.00-131.00)	101.5 (92.25-119.50)	0.321±
TC (mg/dL)	192.43 (41.70)	186.32 (40.23)	198.45 (42.59)	0.067†
TG (mg/dL)	152.94 (36.55)	149.71 (30.09)	156.13 (41.92)	0.269†
LDLc (mg/dL)	141.07 (33.49)	136.36 (32.19)	145.73 (34.30)	0.078†
HDLc (mg/dL)	41.00 (38.00-44.00)	41.00 (39.00-48.00)	39.00 (37.00-42.00)	0.006±
Non-HDLc (mg/dL)	150.46 (42.39)	143.49 (41.61)	157.33 (42.27)	0.039†
TC/HDLc ratio	4.72 (1.31)	4.47 (1.28)	4.97 (1.29)	0.014†
LDLc/HDLc ratio	3.49 (1.10)	3.28 (1.03)	3.69 (1.13)	0.017†
**Adiposity indicators**								
BMI (kg/m²)	26.04 (22.19-30.39)	24.51 (210.8-27.25)	28.39 (22.60-32.94)	0.002±
WC (cm)	92.35 (10.25)	94.20 (10.57)	90.53 (9.64)	0.023†
WHtR	0.58 (0.07)	0.57 (0.07)	0.59 (0.06)	0.044†
C index	1.32 (0.12)	1.35 (0.11)	1.29 (0.11)	0.001†
LAP (cm.mmol.L)	53.79 (24.04)	49.93 (22.54)	57.60 (24.99)	0.044†
VAI	3.08 (1.07)	2.60 (0.80)	3.54 (1.10)	<0.001†
ABSI (m 11/6 kg-2/3)	0.09 (0.08-0.09)	0.09 (0.08-0.09)	0.08 (0.07-0.9)	<0.101±

Data presented as mean (standard deviation) values

(† ; Student's t test) or median (interquartile range) values

(± ; Mann-Whitney test).

Abbreviations: TC, total cholesterol; TG, triglycerides; LDLc, low-density lipoprotein cholesterol; HDLc, high-density lipoprotein cholesterol; TC/HDLc, total cholesterol/high-density lipoprotein cholesterol ratio; LDLc/HDLc, low-density lipoprotein cholesterol/high-density lipoprotein cholesterol ratio; BMI, body mass index; WC, waist circumference; WHtR, waist-to-height ratio; C index, conicity index; LAP, lipid accumulation product; VAI, visceral adiposity index; ABSI, A body shape index.

In the correlation between adiposity indicators and markers of metabolic disorder (Table 2), TG had a moderate positive correlation with LAP and VAI in the overall sample. Among men, TG had a moderate positive correlation with LAP and a strong positive correlation with VAI, while HDLc had a moderate positive correlation with VAI (p < 0.05). In women, TG had a moderate positive correlation with LAP and VAI (p < 0.05). In contrast, the C index and the ABSI presented a low negative correlation with markers of metabolic disorder among women.

**Table 2:** Correlation between adiposity indicators and markers of metabolic disorder according to sex in older adults (n = 159) seen at a geriatric outpatient clinic in the city of Lagarto (Sergipe, Brazil)

Variables	BMI† (kg/m²)	WC (cm)	WHtR	C index	LAP (cm.mmol.L)	VAI	ABSI† (m 11/6 kg-2/3)
**Total sample**							
Glucose (mg/dL)†	0.041	0.147	0.117	0.090	0.170*	0.159*	0.056
TC (mg/dL)	0.174*	-0.503	0.089	-0.167*	0.256**	0.346**	-0.220**
TG (mg/dL)	0.234**	0.083	0.128	-0.134	0.637**	0.678**	-0.149
LDLc (mg/dL)	0.136	-0.108	0.086	-0.163*	0.111	0.067	-0.212**
HDLc (mg/dL)*†	-0.255**	0.033	-0.166*	0.193*	-0.102	0.352**	0.233**
Non-HDLc (mg/dL)	0.195*	-0.062	0.105	-0.195*	0.265**	0.269**	-0.240**
TC/HDLc ratio	0.283**	-0.059	0.150	-0.230**	0.230**	0.021	-0.300**
LDLc/HDLc ratio	0.239**	-0.092	0.133	-0.215**	0.115	-0.138	-0.291**
**Men**							
Glucose (mg/dL)*†	0.137	0.156	0.232*	0.094	0.183	0.122	0.011
TC (mg/dL)	0.086	0.088	0.194	0.091	0.288**	0.225*	0.039
TG (mg/dL)	0.083	0.159	0.130	0.089	0.608**	0.753**	0.116
LDLc (mg/dL)	0.046	-0.007	0.129	0.075	0.072	-0.030	0.005
HDLc (mg/dL)*†	-0.141	-0.029	-0.086	0.092	-0.013	0.557**	0.180
Non-HDLc (mg/dL)	0.106	0.090	0.202	0.073	0.281*	0.127	0.009
TC/HDLc ratio	0.132	0.063	0.188	0.008	0.203	-0.129	-0.071
LDLc/HDLc ratio	0.096	-0.003	0.132	0.079	0.056	-0.276*	-0.083
**Women**							
Glucose (mg/dL)*†	-0.133	0.062	-0.033	0.195	0.081	0.156	0.204
TC (mg/dL)	0.162	-0.145	-0.088	-0.347**	0.196	0.384**	-0.324**
TG (mg/dL)	0.297**	0.059	0.144	-0.263*	0.655**	0.695**	-0.301**
LDLc (mg/dL)	0.173	-0.165	-0.013	-0.324**	0.107	0.031	-0.301**
HDLc (mg/dL)*†	-0.136	0.083	-0.065	0.167	-0.081	0.438**	0.172
Non-HDLc (mg/dL)	0.193	-0.164	-0.074	-0.385**	0.251	0.293**	-0.364**
TC/HDLc ratio	0.266*	-0.119	0.042	-0.383**	0.208	-0.035	-0.388**
LDLc/HDLc ratio	0.243*	-0.116	0.080	-0.333**	0.112	-0.237*	-0.341**

† Spearman's correlation, all other variables were analyzed with Pearson's correlation.

* p < 0.05;

** p < 0.01.

Abbreviations: BMI, body mass index; WC, waist circumference; WHtR, waist-to-height ratio; C index, conicity index; LAP, lipid accumulation product; VAI, visceral adiposity index; ABSI, A body shape index; TC, total cholesterol; TG, triglycerides; LDLc, low-density lipoprotein cholesterol; HDLc, high-density lipoprotein cholesterol; TC/HDL, total cholesterol/high-density lipoprotein cholesterol ratio; LDL/HDL, low-density lipoprotein cholesterol/high-density lipoprotein cholesterol ratio.

In the overall sample, LAP and WHtR had the largest AUCs, with values above 0.80. However, when analyzed by sex, the best predictors of MetS in men were LAP, followed by WC and WHtR, with values above 0.70 but below 0.80. In women, the best predictors of MetS were LAP, WC, WHtR, and BMI, with AUCs greater than 0.86 (p < 0.05) (Table 3).

**Table 3:** Area under the receiver operating characteristic curve values by sex for adiposity indicators associated with metabolic syndrome in older adults (n = 159) seen at a geriatric outpatient clinic in the city of Lagarto (Sergipe, Brazil)

Obesity markers	Total sample		Men		Women	
	Area under the ROC curve	95% CI	Area under the ROC curve	95% CI	Area under the ROC curve	95% CI
BMI (kg/m²)	0.780	0.701-0.860	0.730	0.615-0.845	0.865	0.735-0.978
WC (cm)	0.732	0.637-0.826	0.774	0.667-0.880	0.927	0.863-0.992
WHtR	0.809	0.728-0.889	0.765	0.657-0.873	0.914	0.841-0.986
C index	0.517	0.419-0.615	0.639	0.514-0.765	0.513	0.331-0.696
LAP (cm.mmol.L)	0.821	0.744-0.898	0.781	0.676-0.886	0.933	0.971-0.994
VAI	0.655	0.562-0.748	0.500	0.370-0.630	0.651	0.449-0.853
ABSI (m 11/6 kg-2/3)	0.417	0.320-0.514	0.531	0.400-0.662	0.401	0.213-0.590

Abbreviations: 95% CI, 95% confidence interval; BMI, body mass index; WC, waist circumference; WHtR, waist-to-height ratio; C index, conicity index; LAP, lipid accumulation product; VAI, visceral adiposity index; ABSI, A body shape index.

The optimal cutoff values, the Youden's index, and the respective sensitivity and specificity values of the indicators are presented in Table 4. In the overall sample, LAP and WHtR presented the highest Youden's index values, identifying a cutoff value of 41.73 (sensitivity 85.3%, specificity 69.8%) for LAP and 0.58 (sensitivity 71.6%, specificity 81.4%) for WHtR. When analyzed by sex, LAP, WC, and WHtR presented the highest Youden's index values to predict MetS in women, with cutoff values of 46.82 (sensitivity 85.9%, specificity 77.8) for LAP, 87.0 cm (sensitivity 94.4%, specificity 77.8%) for WC, and 0.58 (sensitivity 88.7%, specificity 66.7%) for WHtR, with PPVs of 93.5%, 97.1%, and 95.5%, respectively. Among men, only LAP presented Youden's index values > 0.50 for the prediction of MetS.

**Table 4:** Cutoff values, Youden's index, sensitivity, and specificity results of indicators predictive of metabolic syndrome categorized by sex among older adults (n = 159) seen at a geriatric outpatient clinic in the city of Lagarto (Sergipe, Brazil)

Indicators		Optimal cutoff value	Youden's index	Sensitivity	Specificity	PPV	NPV
BMI (kg/m²)							
	Total sample	22.91	0.485	81.0	67.4	87.0	56.9
	Men	22.91	0.469	82.2	64.7	75.5	73.3
	Women	23.53	0.649	76.1	88.9	98.2	32.0
WC (cm)							
	Total sample	87.50	0.455	82.8	62.8	85.7	57.4
	Men	89.30	0.477	88.9	58.8	74.1	80.0
	Women	87.00	0.775	94.4	77.8	97.1	63.6
WHtR							
	Total sample	0.58	0.529	71.6	81.4	91.2	51.4
	Men	0.57	0.424	68.9	73.5	77.5	64.1
	Women	0.58	0.761	88.7	66.7	95.5	42.9
C index							
	Total sample	1.33	0.113	50.9	60.5	77.7	31.3
	Men	1.33	0.314	75.6	55.9	69.4	63.3
	Women	1.25	0.214	56.3	22.2	85.2	6.0
LAP (cm.mmol.L)							
	Total sample	41.73	0.551	85.3	69.8	88.4	63.8
	Men	41.36	0.514	86.7	64.7	76.5	78.5
	Women	46.82	0.775	85.9	77.8	93.5	35.9
VAI							
	Total sample	2.54	0.291	73.3	55.8	81.8	43.6
	Men	2.54	0.129	51.5	61.8	59.0	45.0
	Women	3.24	0.341	56.3	77.8	95.3	18.3
ABSI (m11/6kg-2/3)						
	Total sample	0.083	0.221	50.0	72.1	82.9	34.8
	Men	0.087	0.114	55.6	55.9	62.5	48.7
	Women	0.083	0.329	66.2	66.7	94.0	19.9

Abbreviations: PPV, positive predictive value; NPV, negative predictive value, BMI, body mass index; WC, waist circumference; WHtR, waist-to-height ratio; C index, conicity index; LAP, lipid accumulation product; VAI, visceral adiposity index; ABSI: A body shape index.

## DISCUSSION

In older women, the adiposity indicators LAP, WC, and WHtR had the greatest ability to identify MetS. All three indicators had similar performances in identifying MetS, with the highest AUC and Youden's index values in the sample. These are relevant results, considering that these indicators proved to be good predictors for MetS in older women and may become better performing alternative indicators. The performance of the indicators was not satisfactory among men.

The cutoff value for LAP in the overall sample was 41.73. In some studies evaluating LAP, the corresponding cutoff values ranged from 31.6 to 53.63 (6,25–28). However, differences in populations, ethnicities, and age groups among the studies preclude comparison of the results and reveal a lack of agreement among these indicators in predicting MetS. Additionally, LAP is calculated using WC and TG values, which are also diagnostic components of MetS, ensuring a more effective marker.

Even though the equation for LAP calculation is more robust compared with the equations used for other indicators such as WC, BMI, and WHtR, LAP reflects not only visceral fat deposits but also increased lipolytic activity within this compartment of the adipose tissue, resulting in the changes in lipids, glucose, anthropometric measurements, and hemodynamic parameters that characterize MetS (29). This observation underscores the importance of screening for MetS indicators in clinical practice, which can be achieved easily and at a low cost.

For WHtR, the ideal cutoff value identified for women in the present study was 0.58. This contrasts with values obtained in other studies carried out in older adults (6,30–32). The mechanisms by which increased WHtR identifies individuals with MetS are still unclear. Since the WHtR formula uses height values, it bypasses the limitations of WC and averts a potential confusion with height in cardiovascular risk and MetS (33). Considering this adjustment by height (34) and independence from age, WHtR is a potentially advantageous measurement. In a systematic review, Corrêa and cols. (35) described WHtR as a valid index in diagnosing obesity in older adults and a good indicator in predicting risk factors and cardiovascular diseases, diabetes, and MetS when compared with BMI, WC, WHR, and other parameters, in addition to being a more effective individual and collective follow-up parameter in clinical practice.

Aligned with the present findings, other studies (3,6) have also identified WC and BMI as indicators of MetS in older adults. The cutoff values for WC in women in the present study were similar to those recommended by the National Cholesterol Education Program – Adult Treatment Panel III (NCEP-ATP III) and the Brazilian Guideline for the Diagnosis and Treatment of Metabolic Syndrome (2,36). We emphasize that the MetS criteria adopted in the present study (22) do not require the presence of increased WC for the diagnosis of MetS; therefore, not all older adults with MetS in the present study had abdominal obesity. However, the mean WC values in the overall sample and in each sex were higher than the cutoff values for the adopted criteria (≥ 80 cm women and ≥ 90 cm for men in South American populations).

Regarding the C index, the results found were already expected since this index relates more to cardiovascular risk (16). Our results are aligned with those published by Oliveira and cols. (9) and Morais and cols. (3). Even though the C index performs well in the assessment of cardiovascular risk, the same is not true in terms of the prediction of risk of metabolic abnormalities (37).

The performance of ABSI was not satisfactory in the present study. The authors consider this indicator controversial in terms of predicting MetS; this indicator also lacks specific cutoff values for older adults, hindering its use in population studies (7) or even in clinical practice since it involves a complex equation.

Regarding VAI, Amato and cols. (20) have reported that a cutoff value greater than 2.00 (sensitivity 68.5%, specificity 76.0%) is able to predict MetS in individuals older than 66 years. On the other hand, Ejike (27) identified a cutoff value for VAI of 4.4, reporting better sensitivity (67%) and specificity (84%), but without significant AUC values. Although these studies reported greater predictive values than the present study, the results cannot be compared since they were not stratified by sex, and the studies used different diagnostic criteria for MetS. In addition, most studies assessing VAI were conducted in children, adolescents, and adults, limiting the use of cutoff values for older adults.

The different performances of the indicators in men and women may be due to physiological differences between sexes in terms of height, weight, and body composition, mainly reflected by differences in the progressive qualitative and quantitative reduction in skeletal muscle mass and progressive increase in adipose tissue. Men present abdominal and intra-abdominal fat deposits, while women store more fat in the gluteal-femoral region due to hormonal differences in both sexes (32,38).

The present study enabled the identification of specific cutoff values for individuals aged 60 years and older both in the sample as a whole and stratified by sex. This allowed for a better understanding of the performance of each indicator, corroborating and contributing to other findings in the literature, and presenting new knowledge on the prevention of MetS in the older population.

This study has some limitations. Since it was conducted in older adults treated at an outpatient clinic, the data cannot be extrapolated for older adults in the community. In addition, the data were not analyzed by age group, which may have limited the assessment of the performance of each indicator with advancing age. Thus, future studies are needed to assess possible changes in the cutoff values adopted for older adults. In order to determine new criteria and guidelines for MetS and cardiovascular risk, the evaluation criteria should be stratified according to sex and age to assess the influence of body composition and particularities that occur with aging.

In conclusion, the indicators LAP, WC, and WHtR performed well in identifying the occurrence of MetS in older women and may be used in clinical practice since these are simple, low-cost, non-invasive measurements. These results support the early and continuous use of adiposity indicators in older adults since they are accessible, reproducible, sensitive, specific, and reliable. This ensures better prospects for the promotion, prevention, and treatment of MetS in older adults.
